# Outcome Predictors of Stroke Mortality in the Neurocritical Care Unit

**DOI:** 10.3389/fneur.2020.579733

**Published:** 2020-12-15

**Authors:** Dmitriy Viderman, Alpamys Issanov, Talgat Temirov, Ewan Goligher, Philip la Fleur

**Affiliations:** ^1^Nazarbayev University School of Medicine (NUSOM), Nur-Sultan, Kazakhstan; ^2^National Research Oncology Center, Nur-Sultan, Kazakhstan; ^3^Astana Medical University, Nur-Sultan, Kazakhstan; ^4^Division of Respirology, Department of Medicine, University Health Network, Toronto, ON, Canada; ^5^Interdepartmental Division of Critical Care Medicine, University of Toronto, Toronto, ON, Canada; ^6^Toronto General Hospital Research Institute, Toronto, ON, Canada

**Keywords:** cerebrovascular disease, intensive care unit, critical care, mortality, risk factors, hemorrhagic stroke, ischemic stroke

## Abstract

**Background:** Risk factors for medium to long-term mortality after stroke are well-established but predictors of in-hospital stroke mortality are less clearly characterized. Kazakhstan has the highest age-standardized mortality rate from ischemic stroke in the world.

**Methods:** We performed a retrospective analysis of patients with stroke who were admitted over a 3.5-years period to the neurocritical care unit of a tertiary care hospital in Nur-Sultan, Kazakhstan.

**Results:** In total, 148 critically ill patients were included in the analysis (84 ischemic stroke, 64 hemorrhagic stroke). The mean age was 63 years, 45% were male and the mean Glasgow Coma Score (±*SD*) at baseline was 10.3 (±3.4). The in-hospital mortality rate was similar in patients with ischemic (36%) and hemorrhagic (39%) stroke (HR 0.88, 95%CI 0.48–1.60). Median survival was 38 days (range: 1–89 days) in patients with ischemic stroke and 39 days (range: 1–63 days) in patients with hemorrhagic stroke. Univariable analysis found that patients who had a lower Glasgow Coma Scale, were in coma and who had cerebral edema were more likely to die in-hospital (*P* = 0.04, 0.02, <0.01, respectively).

**Conclusions:** Our analysis showed that mortality risk in critically ill patients with hemorrhagic stroke was closer to mortality risk in patients with ischemic stroke than has been reported in other analyses. Hypertension, chronic heart failure, ischemic heart disease and atrial fibrillation were the most frequent comorbidities in patients who developed severe (life-threatening) stroke. Coma and cerebral edema on admission appear to be associated with poor outcome. This is the first publication of in-hospital stroke mortality from a Central Asian population and could form the basis for future research including development of risk scores and identifying modifiable risk factors.

## Background

Stroke remains the second most common cause of death and the predominant cause of disability worldwide despite recent advances in its management (1, 2). Stroke-related complications most frequently occur during the 1st week after stroke and are associated with higher mortality risk during hospital stay and increased length of hospital stay (3–5). Admissions to intensive care units for stroke are increasing and this trend can be expected to continue in aging populations (6). Intensive care units are extremely important for management of critically ill stroke patients and admission criteria usually include single or multiple organ dysfunction/failure, low Glasgow Coma Scale score, necessity for advanced monitoring and postoperative observation.

The prognosis is generally considered to be poor for stroke patients admitted to critical care units. Short term in-hospital mortality rates as high as 50% (7–9). Currently available therapeutic options are limited and even if patients survive the acute phase and are discharged from intensive care, persistent paralysis and cognitive dysfunction are common and require long-term rehabilitation. Therefore, the identification of risk factors for poor outcomes in critically ill stroke patients could improve patient management and allow for more precise prognosis estimation. Factors that have previously been identified as predictors for in-hospital mortality in other studies include age, localization of ischemia, National Institute of Health Stroke Scale (NIHSS) score, Acute Physiology and Chronic Health Evaluation score (APACHEII), Glasgow Coma Scale (GCS) score, and the Simplified Acute Physiology Score (SAPS) (9). The main goal of neurocritical care is directed toward prevention, early recognition and management of secondary brain damage that includes cerebral edema, intracranial hypertension, impaired cerebral circulation, hypotension, hypertension, hypoxia, hypercapnia, and hyperthermia. Therefore, the identification of modifiable risk factors in critically ill stroke patients could improve patient outcomes.

Until recently, very little has been known about the prevalence of modifiable and non-modifiable risk factors for cerebrovascular disease in Central Asia. Kazakhstan has the highest age-standardized mortality rate from ischemic stroke in the world (149–174 deaths per 100,000 person years) (10). Recent cross-sectional surveys have indicated that the prevalence of dyslipidemia, diabetes and hypertension is high in this region (11–13). Therefore, in this study, we aimed to identify prognostic factors for in-hospital mortality in critically ill Central Asian patients with acute ischemic or hemorrhagic stroke.

## Methods

We performed a retrospective analysis of routinely collected data from patients diagnosed with ischemic or hemorrhagic stroke at the University Medical Center in Nur-Sultan, Kazakhstan. We included all adults (age >18 years) who were admitted to the neurocritical care unit from October 2009 to March 2013.

All patients presented to our hospital with stroke underwent a standardized workup by a neurologist and stroke diagnosis was confirmed by computed tomography or magnetic resonance tomography. The management of acute stroke was conducted in accordance with our local stroke management protocol based on the American Heart Association/American Stroke Council recommendations (14, 15).

In our center, criteria used to determine admission to the neurocritical care unit included impaired consciousness (GCS ≤ 12); need for mechanical ventilation (apnoea, ataxic or cluster breathing), respiratory failure (tachypnoea, dyspnoea at rest, bradypnoea, hypopnoea, apnoea, ataxic, or cluster breathing, use of accessory muscles), inability to protect airway, clinically significant brain edema, evidence of raised intracranial pressure, monitoring required (level of consciousness, respiratory function, intracranial pressure, and continuous electroencephalography) and post-neurosurgical intervention.

This study was approved by two local Ethics Committees (Nazarbayev University and the University Medical Center). Patient consent was not requested because the study was retrospective and data were extracted excluding personal identifiers.

### Statistical Analysis

Descriptive analyses on normally distributed continuous variables were performed by calculating means and standard deviations while medians and interquartile ranges for non-normally distributed numeric variables. Frequencies and percentages were summarized for categorical variables. Bivariable analysis by stroke type was done using the chi-squared test or Fisher exact test for categorical variables, and independent *t*-test with equal variances for continuous variables. To examine the survival probabilities by stroke type and by presence of coma signs on admission, Kaplan Meier estimators with log rank test were used. Since one of the main predictors—history of hypertension—was highly associated with the outcome variable (quasi-separation) this caused numerical issues when calculating the hazard ratio in Cox proportional hazards regression analysis. Therefore, we used survival analysis with Firth's penalized estimation to calculate crude and adjusted hazard ratio estimates (16). To analyze statistical differences in repeated measurements of the Glasgow Coma Scale and arterial blood pressure over time between categories and the interaction between time and categories, we used two-way ANOVA with repeated measurements. Statistical analysis was performed using STATA (version 15.1, StataCorp USA) and R (version 3.5.3) statistical software.

## Results

### Characteristics of Patients at Admission

One hundred and forty eight patients were admitted to the neurocritical care unit at our center for stroke between October 2009 and March 2013. The most common co-morbidities were hypertension, ischemic heart disease, chronic heart failure and atrial fibrilation (Table 1). Most of the patients were admitted directly to neurocritical care upon presentation to the emergency department of our center (127/148 [85.8%]) or during the 1st day after presentation to our center (11/148 [7.4%]). The remaining 10 (6.8%) patients were transferred to neurocritical care between day 2 and 14 after admission to the hospital. Eighty four of 148 patients (56.8%) were diagnosed with ischemic stroke and 64/148 (43.2%) were diagnosed with hemorrhagic stroke (Table 1). Of the patients with ischemic stroke, 56/84 (66.7%) had middle cerebral artery occlusion and 27/84 (32.1%) had vertebrobasilar artery occlusion. Mean Glasgow Coma Scale scores were higher at admission in patients with ischemic stroke, compared to patients with hemorrhagic stroke (Table 1).

**Table 1:** Clinical characteristics of patients with stroke admitted to neurocritical care.

**Variable**	**Total, *N* = 148 (100%)**	**Ischemic stroke, *n* = 84 (56.8%)**	**Hemorrhagic stroke, *n* = 64 (43.2%)**
Mean age^*^±*SD*, years	63.3 ± 14.2	68.8 ± 11.4	56.2 ± 14.4
Male	66 (44.6)	37 (44.0)	29 (45.3)
Arterial hypertension	139 (93.9)	80 (95.2)	59 (92.2)
Ischemic heart disease^*^	57 (38.5)	44 (52.4)	13 (20.3)
Chronic heart failure	88 (59.5)	55 (65.5)	33 (51.6)
Diabetes mellitus^*^	29 (19.6)	23 (27.4)	6 (9.4)
Atrial fibrillation^*^	33 (22.3)	29 (34.5)	4 (6.3)
Mean SBP ±*SD*, mmHg^*^	171.1 ± 40.1	162.8 ± 32.4	181.8 ± 46.5
Mean heart rate ±*SD*, bpm	87.8 ± 15.9	87.6 ± 16.9	88.2 ± 14.6
Mean GCS at admission ±SD^*^	10.3 ± 3.4	11.0 ± 3.3	9.3 ± 3.4
**GCS score**			
<9	58 (39.2)	25 (29.8)	33 (51.6)
9–11	20 (13.5)	12 (14.3)	8 (12.5)
>11	70 (47.3)	47 (55.9)	23 (35.9)
Coma^*^	58 (39.2)	25 (29.8)	33 (51.6)
Cerebral edema^*^	63 (42.9)	26 (31.3)	37 (57.8)

*Numbers are n(%) unless otherwise specified*.

* *Ischemic stroke vs. hemorrhagic stroke, p < 0.05*.

Out of 148 patients, 58 (39.1%) were in coma at time of admission to neurocritical care and had a Glasgow Coma Scale <9 points. A smaller proportion of the patients with ischemic stroke were in coma (30%) compared to patients with hemorrhagic stroke (52%). Patients with ischemic stroke were, on average, older than patients with hemorrhagic stroke. History of ischemic heart disease, diabetes mellitus and atrial fibrillation was more common in patients who had an ischemic stroke, relative to patients who had a hemorrhagic stroke (Table 2). Eighteen (12.1%) patients had chronic renal failure. History of arterial hypertension was common (94%) with mean systolic blood pressure (±*SD*) at admission of 171(±40) mmHg and mean diastolic blood pressure at admission of 95(±15) mmHg; however, only 105 (71%) patients were taking antihypertensive medications at admission.

**Table 2:** In-hospital complications.

**Event**	**Total, *N* = 148 (100%)**	**Ischemic stroke, *n* = 84 (56.8%)**	**Hemorrhagic stroke, *n* = 64 (43.2%)**
Death (all cause)	55 (37.2)	30 (35.7)	25 (39.1)
Dysrhythmia^*^	43 (29.0)	33 (39.3)	10 (15.6)
Respiratory failure (Stroke induced)^*^	93 (62.8)	46 (54.8)	47 (73.4)
Ventilator-associated pneumonia	32 (21.6)	18 (21.4)	14 (21.9)
Acute renal failure	15 (10.1)	8 (9.5)	7 (10.9)
Gastrointestinal bleeding	10 (6.8)	7 (8.3)	3 (4.7)
Myocardial infarction	9 (6.1)	7 (8.3)	2 (3.1)
Urinary tract infection	6 (4.1)	5 (5.9)	1 (1.6)
Acute heart failure^*^	6 (4.1)	6 (7.1)	0 (0.0)
Liver failure	5 (3.4)	3 (3.6)	2 (3.1)
Re-occurring stroke	4 (2.7)	3 (3.6)	1 (1.6)
Shock	2 (1.4)	2 (2.4)	0 (0.0)

* *Ischemic vs. hemorrhagic stroke, p < 0.05*.

### Patient Outcomes

The median duration (interquartile range) of hospital stay was 12 days (6–22.5 days) and the median neurocritical care unit stay was 7 days (range: 1–68 days). Neurosurgical procedures were performed in 27 (18%) patients. One patient with ischemic stroke underwent decompression craniotomy and 26 (41%) patients with hemorrhagic stroke underwent craniotomy with hematoma removal.

**Figure 1:**
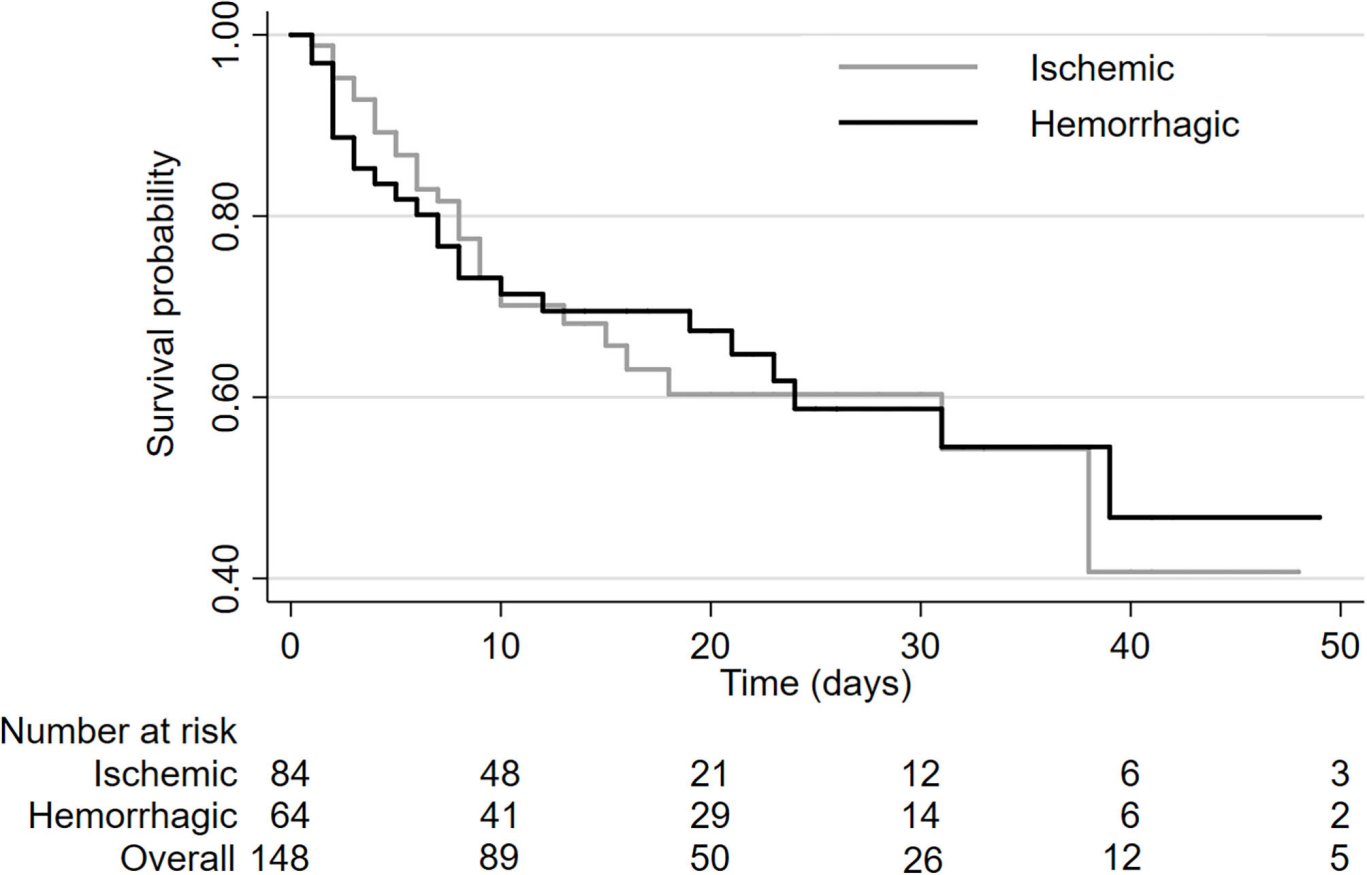
Kaplan-Meier survival analysis by stroke type.

Of the patients with stroke who were admitted to neurocritical care, 55/148 patients (37%) died in hospital. The in-hospital mortality rate was similar in patients with ischemic (36%) and hemorrhagic (39%) stroke (HR 0.88, 95%CI 0.48–1.60, log-rank *P* = 0.99; Figure 1). Median survival was 38 days (range: 1–89 days) in patients with ischemic stroke and 39 days (range: 1–63 days) in patients with hemorrhagic stroke.

Respiratory complications were the most common extra-cerebral complications and 93 (63%) patients developed stroke-related respiratory failure (coma, loss of airway protective reflexes, reduced minute ventilation, pneumonia, and acute lung injury) requiring intubation with mechanical ventilation (Table 2). Respiratory failure was less common in patients with ischemic stroke (55%) than in patients with hemorrhagic stroke (73%). Hemorrhagic transformation of stroke occurred in three patients.

### Predictors of In-Hospital Mortality

Univariable analysis revealed several characteristics that were correlated with mortality rate (Table 3). All patients in our analysis were admitted to neurocritical intensive care. At admission, patients who had a lower Glasgow Coma Scale, were in coma and who had cerebral edema were more likely to die in-hospital (*P* = 0.05, 0.02, < 0.01, respectively). Statistically significant variables from the univariable analysis and clinically important variables were used in the multivariable analysis (age, sex, hypertension, ischemic heart disease, chronic heart failure, systolic blood pressure, heart rate, presence of cerebral edema, and stroke type). In multivariable analysis, male sex (HR 0.48, 95%CI: 0.22–0.98) and hemorrhagic stroke (vs. ischemic stroke, HR 0.44 95%CI:0.23–1.22) trended toward a lower risk of in-hospital mortality. Cerebral edema was associated with a higher risk of mortality (HR 2.45 95%CI: 1.04–6.13).

**Table 3:** In-hospital stroke mortality predictors using simple and multivariable Cox regression analysis.

**Predictors**	**Unadjusted HR (95% CI)**	***p*-value**	**Adjusted HR (95% CI)**	***p*-value**
Age per 10 years	1.02 (0.81–1.30)	0.85	0.89 (0.63–1.27)	0.53
**Gender**				
Female	Reference	0.94	Reference	0.04
Male	0.60 (0.31–1.12)		0.48 (0.22–0.98)	
**History of hypertension**				
No	Reference	0.18	Reference	0.09
Yes	5.15 (0.56–688.5)		8.71 (0.77–1229.4)	
**History of IHD**				
No	Reference	0.29	Reference	0.33
Yes	1.42 (0.74–2.76)		1.48 (0.67–3.34)	
**CHF**				
No	Reference	0.81	Reference	0.37
Yes	0.92 (0.48–1.77)		0.70 (0.32–1.52)	
**History of diabetes**				
No	Reference	0.14		
Yes	0.47 (0.15–1.26)			
**History of atrial fibrillation**				
	Reference	0.73		
Yes	1.13 (0.54–2.27)			
SBP per 10 mm Hg	1.03 (0.94–1.13)	0.52	1.01 (0.91–1.12)	0.81
HR per 10 beat/min	1.10 (0.91–1.33)	0.30		
GCS per one unit^*^	0.91 (0.82–1.00)	0.05	0.96 (0.85–1.09)	0.56
**Coma**				
No	Reference	0.02		
Yes	2.10 (1.12–3.97)			
**Cerebral edema**				
No	Reference	<0.01	Reference	0.04
Yes	3.17 (1.51–7.10)		2.45 (1.04–6.13)	
**Type of stroke**				
Ischemic	Reference	0.67	Reference	0.14
Hemorrhagic	0.88 (0.48–1.60)		0.55 (0.23–1.22)	

## Discussion

The lifetime risk of ischemic or hemorrhagic stroke in adults is 25% and the risk of ischemic stroke is 18% from the age of 25 years onward (17). Estimates for the incidence of stroke range from 90 to 180 per 100,000 population in North America and Western Europe to 241–360 per 100,000 population in East Asia, Eastern Europe and Central Asia (18). Recent epidemiologic data suggest that the highest incidence of stroke is in East Asia, followed by Eastern Europe and Central Asia (18). Data from the Global Burden of Ischemic and Hemorrhagic Stroke (GBIHS) study indicate that while Kazakhstan has a low prevalence of ischemic stroke (0–170 cases per 100,000 person years), it has the highest age-standardized mortality rate from ischemic stroke in the world (149–174 deaths per 100,000 person years) (10). Age standardized prevalence of hemorrhagic stroke is 39–78 cases per 100,000 person-years. Mortality due to hemorrhagic stroke is 64–95 deaths per 100,000 person-years, a rate which is higher than developed countries and higher than most developing countries.

In our study, mortality was high because we included only severely ill patients. The unadjusted rates of mortality were similar for patients with ischemic and hemmorhagic stroke, but in the multivariable analysis male sex was protective. In comparison to ischemic stroke, hemmorhagic stroke was associated with reduced risk of death and this is consistent with the GBIHS study.

Overall rate of in-hospital mortality was similar in patients with ischemic stroke compared to patients with hemorrhagic stroke despite the fact that mean age was higher amongst patients with ischemic stroke (mean age 68.8 years) compared to patients with hemorrhagic stroke (mean age 56.2 years). A majority of the deaths occurred within the first 10 days after admission and deaths tended to occur earlier in patients with ischemic stroke, though this did not reach statistical significance in the multivariable analysis (HR 0.55, 95%CI 0.23–1.22; *p* = 0.14; Figure 1). Hemorrhagic stroke was associated with a slightly longer length of hospital stay. While it would be expected that patients with hemorrhagic stroke would have a higher in-hospital mortality risk, we did not observe this in our analysis. This could be a spurious finding because of the sample size was not large. It could also be a related to the older age of our patients and a high prevalence of comorbidities (atrial fibrillation, ischemic heart disease, hypertension, and diabetes) in ischemic stroke patients.

The univariable analysis identified coma and cerebral edema as the most strongly correlated risk factors for subsequent in-hospital mortality (Table 2). None of the modifiable risk factors showed a statistically significant impact on mortality in the multivariable model, but history of hypertension had a large hazard ratio with high statistical uncertainty [HR(95%CI) 8.71(0.77–1,229)]. The prevalence of hypertension in our study is higher than a recent study performed in the same city which showed a prevalence of at 70% in adults aged 50–75 years (11). Recent studies reported that hypertension is associated with unfavorable outcome in patients with acute ischemic stroke (19). Another study showed that hypertension was correlated with death and disability in patients with hemorrhagic stroke but not with ischemic stroke in Inner Mongolia (20).

### Gastrointestinal Complications

All patients were given an acid-suppressive therapy (proton pump inhibitor or H2-blocker), however, 10 patients (7%) still developed gastrointestinal bleeding. Gastrointestinal bleeding was more common in patients with ischemic stroke (*N* = 7) and this difference was likely due to the use of antiplatelet medications in this group. Nine cases of GI bleeding were successfully treated either medically or endoscopically and there was only one severe case which required surgery. All patients who developed bleeding were in coma and were mechanically ventilated. GI bleeding is a well-documented complication of acute phase of stroke with a reported incidence of 0.1–8.0% (21, 22).

Mucosal injury is extremely common in ICU patients and was reported to be 75–100% in those who were admitted in shock (23). Sympathetic drive and vasoconstriction in neurosurgical patients is a probable causative mechanism of the high incidence of gastric ulcers (24) and although a high proportion of critically ill patients develop mucosal injury, not all of these progress to gastrointestinal bleeding (23). Another potential mechanism leading to stress ulcer is reduced mucosal circulation and mucosal hypoxia (25). The impact of acid suppressing medication in stroke patients deserves further study to identify patients who could benefit most. Several meta-analysis have been published to date but the results are inconclusive. Acid suppression strategies have not demonstrated a mortality benefit (26, 27).

### Cardiovascular Complications

Central nervous system pathology has been clearly associated with myocardial damage and dysfunction (28). The mechanism of myocardial injury in acute ischemic stroke is not well-understood but elevated serum catecholamine level has been hypothesized to be a causative factor (29). Conversely, the autoregulation of cerebral blood flow in ischemic stroke is reduced and depends on cardiac function, therefore this can result in vicious cycle of neurocardic dysfunction (30–32).

We found that 8.3% of patients with ischemic stroke and 3.1% of patients with hemorrhagic stroke developed myocardial infarction. Patients with ischemic stroke were more likely to develop myocardial infarction likley related to advanced ischemic heart disease. Previous studies have shown that myocardial infarction is a probable complication in ischemic stroke patients and that this may be related to release of endogenous catecholamines (33).

A strength of this study is the wide range of risk factors that were assessed in a population not previously studied and idenfied several predictors of mortality in this Central Asian population. The main limitation of this study is that it was a retrospective analysis of a small sample. We did not have data on the time from stroke onset to presentation at our center. Thrombolytic therapy with recombinant tissue plasminogen activator was uncommonly used in Kazakhstan during the patient enrollment period (2009–2013) because of access and the likelihood of presentation to hospital more than 4.5 h after stroke onset, therefore, none of the patients in our study received this intervention.

## Conclusion

This is the first analysis of its type in Central Asian patients with stroke, providing both a descriptive element and estimates of the relative contributions to mortality of several risk factors. Our analysis showed that mortality risk in patients with hemorrhagic stroke was closer to mortality risk in patients with ischemic stroke than presented in other analyses. Whether this was an artifact of the sample reflects a regional difference needs to be clarified. Hypertension, chronic heart failure, ischemic heart disease and atrial fibrillation were the most frequent comorbidities in patients who developed severe (life-threatening) stroke. Coma and cerebral edema on admission appear to be associated with poor outcome. Future research directions could include pooled analyses from multiple centers in the region to identify risk factors for in-hospital stroke mortality with greater precision.

## Data Availability Statement

The dataset will be available upon request. Requests to access these datasets should be directed to dmitriy.viderman@nu.edu.kz.

## Ethics Statement

This study was reviewed and approved by the Nazarbayev University IREC. Written informed consent for participation was not required for this study in accordance with the national legislation and the institutional requirements.

## Author's Note

This study was performed at the University Medical Center.

## Author Contributions

DV: conception, design of the study, interpretation of data, drafting the manuscript, manuscript revision, and administrative support. AI: interpretation, statistical analysis, data management, and manuscript revision. TT: administrative support. EG: manuscript revision. PF: obtaining funding, design of the study, interpretation of data, drafting the manuscript, and manuscript revision. All authors contributed to the article and approved the submitted version.

## Conflict of Interest

The authors declare that the research was conducted in the absence of any commercial or financial relationships that could be construed as a potential conflict of interest.
